# Assessing the Economic Benefit of Mass COVID-19 Vaccination Program in Iran: A Real-World Modeling Study

**DOI:** 10.34172/ijhpm.8852

**Published:** 2025-10-11

**Authors:** Hamidreza Jamaati, Saeed Karimi, Yunes Panahi, Shahnam Arshi, Maryam Hajimoradi, Fatemeh Sadat Hosseini-Baharanchi, Fariba Ghorbani, Seyed Mohsen Zahraei, Fatemeh Nouri, Ali Akbari Sari, Mahshad Goharimehr, Abdolreza Mohamadnia, Payam Tabarsi, Farzaneh Dastan, Babak Sharif-Kashani, Majid Marjani, Farin Rashid Farokhi, Seyed Mohammad Reza Hashemian, Mostafa Noorizadeh, Mojtaba Nouhi, Katayoun Tayeri, Sima Noorali, Farnaz Ahmadi, Makan Sadr, Azadeh Moradkhani, Mahdi Ahmadinia, Bahamin Astani, Rajabali Daroudi, Shadi Shafaghi

**Affiliations:** ^1^Chronic Respiratory Disease Research Center, National Research Institute of Tuberculosis and Lung Disease, Shahid Beheshti University of Medical Science, Tehran, Iran.; ^2^Department of Ophthalmology, Torfeh Medical Center, Shahid Beheshti University of Medical Sciences, Tehran, Iran.; ^3^Chemical Injuries Research Center, Baqiyatallah University of Medical Sciences, Tehran, Iran.; ^4^Center for Communicable Disease Control, Ministry of Health and Medical Education, Tehran, Iran.; ^5^Lung Transplantation Research Center, National Research Institute of Tuberculosis and Lung Diseases (NRITLD), Shahid Beheshti University of Medical Sciences, Tehran, Iran.; ^6^Department of Biostatistics, School of Public Health, Iran University of Medical Sciences, Tehran, Iran.; ^7^Tracheal Diseases Research Center, National Research Institute of Tuberculosis and Lung Diseases (NRITLD), Shahid Beheshti University of Medical Sciences, Tehran, Iran.; ^8^Department of Health Management, Policy and Economics, School of Public Health, Tehran University of Medical Sciences, Tehran, Iran.; ^9^Clinical Tuberculosis and Epidemiology Research Center, National Research Institute for Tuberculosis and Lung Disease (NRITLD), Shahid Beheshti University of Medical Sciences, Tehran, Iran.; ^10^Department of Cardiology, Lung Transplantation Research Center, National Research Institute of Tuberculosis and Lung Diseases (NRITLD), Shahid Beheshti University of Medical Sciences, Tehran, Iran.; ^11^Chronic Kidney Disease Research Center, Shahid Beheshti University of Medical Sciences, Tehran, Iran.; ^12^Department of Pharmaceutical Biotechnology, Faculty of Pharmacy, Bulent Ecevit University, Zonguldak, Turkey.; ^13^Department of Biotechnology, Islamic Azad University of Medical Science, Tehran, Iran.; ^14^Health Economic Department, School of Medicine, Shahed University, Tehran, Iran.; ^15^Virology Research Center, National Research Institute of Tuberculosis and Lung Diseases (NRITLD), Shahid Beheshti University of Medical Sciences, Tehran, Iran.

**Keywords:** COVID-19 Vaccination, Cost-Effectiveness, ICER, QALY, Economic Evaluation, Iran

## Abstract

**Background:**

The global challenges posed by COVID-19 vaccinations require careful consideration by decision-makers at both the global and national levels, particularly in developing countries. This study aimed to evaluate the health and economic implications of implementing vaccination programs.

**Methods:**

Two scenarios, one involving vaccination and the other without, were analyzed using the Markov susceptible-infectious-recovered (SIR) model. For the vaccination scenario, real-world data, such as age-specific vaccination coverage, hospitalization rates, and mortality, were obtained from the Medical Care Monitoring Center (MCMC) and national COVID-19 registry during the Omicron wave in Iran. For the counterfactual non-vaccination scenario, we relied on model-based assumptions using published literature and expert input to estimate infection rates and clinical outcomes in the absence of vaccination. The incremental cost-effectiveness ratio (ICER) of the COVID-19 vaccination program was calculated by comparing the incremental cost per unit of quality-adjusted life year (QALY) generated to a willingness-to-pay (WTP) threshold equivalent to 1 time the gross domestic product (GDP) per capita, approximately US$4091. One-way sensitivity analysis was conducted to ensure the reliability of the results.

**Results:**

Overall, 2 098 495 extra QALYs were generated by vaccination, incurring a total extra cost of $853.78 million. The vaccination program resulted in an average of 0.035 incremental QALYs at an additional cost of $14.08 per person. The average ICER for adult vaccination was $406.85 per QALY, indicating that it is a highly cost-effective strategy compared to non-vaccination across all age groups. Vaccinating elderly individuals proved to be the most cost-effective approach among all age categories.

**Conclusion:**

The integrated Markov-SIR model used in this study provides valuable insights into both the health and economic impact of the COVID-19 vaccination program in Iran. These findings support the implementation of vaccination strategies and provide a framework for decision-makers to consider when formulating policies.

## Introduction

**Key Messages**

**Implications for policy makers**

- A key component of decision-making, as well as the execution, assessment, and oversight of public policies, is cost-effectiveness analysis.
- Comparing the vaccination strategy to no vaccination, our findings showed that it significantly decreased the incidence of infections, hospitalizations, intensive care unit (ICU) admissions, and deaths among adults of all ages.
- By raising some additional expenses, expanding the vaccination strategy to eligible individuals would result in a significant increase in quality-adjusted life years (QALYs), making it cost-effective.
- Consequently, the COVID-19 vaccination program in Iran was both extremely cost-effective and beneficial to health.

**Implications for the public**

The cost-effectiveness of Iran’s COVID-19 mass vaccination program during the Omicron era was assessed in this study. A tool that articulates scientific knowledge, empirical evidence, and public policy in a framework for interaction with users, analysts, and decision-makers is our model for evaluating the impact of COVID-19 vaccination strategies. The most economical strategy across all age groups was determined to be immunizing the elderly. In Iran, the COVID-19 vaccination program has been both health-promoting and extremely cost-effective. Therefore, it would be cost-effective to extend the vaccination strategy to eligible individuals, as this would result in a significant increase in quality-adjusted life years (QALYs) while incurring some additional costs.

In November 2019, the severe acute respiratory syndrome coronavirus 2 caused the novel COVID-19, which was officially designated as a public health emergency with international ramifications by the World Health Organization (WHO) 2 months later.^1^ As of April 2023, there have been over 762 million confirmed cases worldwide, resulting in a significant mortality rate of more than 6.8 million people since its initial appearance.^2^ The advent of COVID-19 has been compared to the economic conditions during World War II, which pervaded every aspect of human existence, caused economic fallout, and generated mounting strain on health budgets.^1,3,4^ The solution to effectively reducing the dissemination of COVID-19 was attributed to its vaccine as the ultimate cure.^4,5^ Therefore, there was a major necessity to speed up the development of vaccines at an unparalleled pace and scope.^5^ Authorized vaccines differ in terms of expenses, cold-storage exigencies, clinical effectiveness, and safety characteristics. Consequently, national authorities must choose the most applicable vaccine(s), contingent upon their respective country profiles, outbreak status, and immunization approach.^6^

 Iran’s vaccination program was officially initiated on February 9, 2021, to protect public health and eventually facilitate the resumption of societal engagements. Iranian residents were able to access vaccines at no cost and were obligated to receive 2 doses of the same vaccine. As of February 2023, a majority of Iranian inhabitants (77.58%) had undergone the initial dose of vaccination, whereas 69.7% received the second dose.^2^

 Ensuring adequate allocation of limited financial resources is mandatory for the successful implementation of vaccination strategies, particularly in such circumstances that require immunizing a critical number of individuals, which requires a significant financial scale of finance.^4,7^ Cost-effectiveness analysis is presumed to be a pivotal gateway measure that influences the decision-making process.^8^ In unprecedented circumstances, such as COVID-19, where the availability of accurate data is uncertain and decision-making is time-sensitive, mathematical models may be utilized to estimate the health and economic consequences of healthcare interventions, providing a comprehensive and pragmatic approach. However, in such an emergency, the uncertainty related to the estimated health and economic effects of interventions is high due to the low level of available evidence. Over time, more reliable evidence has been provided regarding the effectiveness and cost of COVID-19 interventions; in addition, there is also real-world evidence regarding these interventions. Therefore, the consequences of COVID-19 interventions, such as vaccination, can be estimated more accurately. In recent years, the use of real-world evidence in the economic evaluation of health interventions has increased.^9^

 In low- and middle-income countries (LMICs), it is crucial to realize the immediate and prolonged impacts of implementing interventions on national budgets to ensure the program’s long-term viability.^8^ The strategic group of experts on immunization established by the WHO has presented a roadmap intended to guide countries toward prioritizing the distribution of limited doses of immunization, which have predominantly concentrated on many contexts, while less attention has been directed toward LMIC settings.^10,11^ The present study aimed to estimate the health and economic consequences of administering COVID-19 vaccinations in Iran using decision-analytic modeling and real-world data. The findings of this study will help policy-makers evaluate the effects of the COVID-19 vaccination program after its implementation, and can also be useful in the context of LMIC countries.^10,11^ This study addresses the scarcity of context-specific economic evaluations of COVID-19 vaccination in the Middle East, particularly in Iran, by using national registry data to model real-world age-stratified outcomes and inform resource allocation in low-resource settings.

## Methods

###  Study Design

 To estimate the economic value of a COVID-19 vaccination program in Iran, we developed a Markov model that divided the population into 6 age groups: 18-24, 25-34, 35-44, 45-54, 55-64, and 64+ years. We applied a Markov-based susceptible-infectious-recovered (SIR) framework that integrates epidemiological progression with economic evaluation components such as healthcare costs, productivity losses, and quality-adjusted life years (QALYs). This combined structure enabled a comprehensive cost-effectiveness analysis of COVID-19 vaccination strategies. The evaluation of the program’s overall economic value considered both medical and nonmedical costs, such as medical treatment expenses, vaccination costs, productivity losses due to missed workdays, and utility losses of COVID-19 patients in all age categories. To assess the cost-effectiveness of the program, we applied the Markov-SIR model, which has been validated and used in many studies related to COVID-19 prevention interventions.^12-14^ The evaluation was conducted from a societal perspective, assuming a discount rate of 3% annually for future costs and health effects. We constructed the model using TreeAge Pro 2020 and reported our analysis according to the Consolidated Health Economic Evaluation Reporting Standards 2022 statement. The Markov-based SIR structure applied in this study does not allow for reinfection, which is a known limitation of classical SIR models. However, due to the short one-year horizon and national data indicating low reinfection rates during the Omicron wave in Iran, this assumption was considered acceptable. To address this uncertainty, the infection rate parameter was varied in one-way sensitivity analyses to indirectly account for the potential effect of reinfection. The 3% annual discount rate was selected based on standard recommendations from the WHO and the US Panel on Cost-Effectiveness in Health and Medicine.^15,16^

###  Model Structure

**Figure 1:**
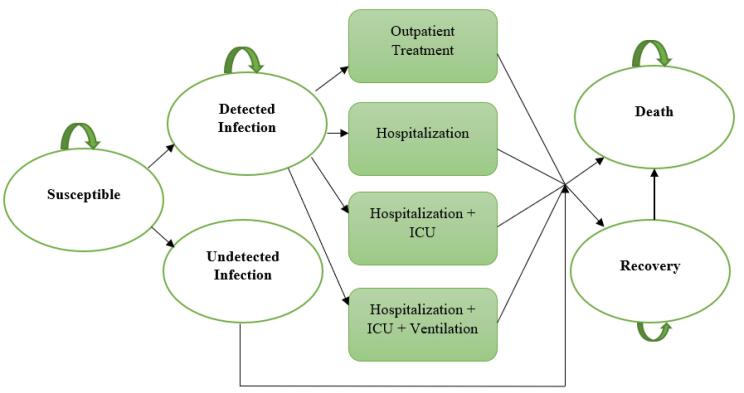
Markov Model of COVID-19 Disease Progression. Abbreviation: ICU, intensive care unit.

 The model of COVID-19 infection and progression is illustrated in Figure 1, which depicts the various states that individuals can move into during each monthly cycle. These states include detected infection, undetected infection, non-infected, or death, and individuals can remain in their current state, as indicated by the arrows. Although the model is conceptually grounded in a SIR framework, it was adapted to include clinical disease progression states such as outpatient care, hospitalization, intensive care unit (ICU) admission, and death. These transitions reflect real-world treatment pathways and allow for health economic evaluation within a Markov structure.

 Patients in the detected infection state only remain there for one cycle, during which they enter a probability tree that allocates them through various levels of COVID-19 treatment, outpatient treatment, hospitalization, hospitalization + ICU admission, and hospitalization + ICU admission + mechanical ventilation to their final resolution of either recovery or death. Non-infected and recovered individuals remain in their states until they die of causes other than COVID-19 infection.

 To assess the cost-effectiveness of vaccination compared to a non-vaccination scenario for each age group, we developed 2 hypothetical cohorts and followed them independently for their lifetime. The vaccination scenario includes partial vaccination, representing individuals who have received only 1dose of the vaccine, and full vaccination, representing individuals who have received at least 2 doses of the vaccine. We assumed that individuals can only contract COVID-19 during the first year of the model and that the vaccine’s effectiveness lasts up to 1 year. Additionally, we assumed that no reinfection occurs within the one-year period.

###  Model Parameters 

####  Transition Probabilities

 The model utilized COVID-19-related transition probabilities, which encompassed various stages of the disease. These probabilities included the monthly infection rate of symptomatic individuals, the likelihood of hospitalization for those infected, the probability of ICU admission for hospitalized patients, the possibility of mechanical ventilation for those admitted to the ICU, and the likelihood of death in all these categories. The data are shown in Table S1 (Supplementary file 1).

 Due to the lack of reliable age-specific data on symptomatic infection rates among unvaccinated individuals during the Omicron wave in Iran, we employed an indirect estimation method commonly used in epidemiological modeling, particularly during the COVID-19 pandemic. Specifically, we divided the observed age-specific hospitalization rates for unvaccinated individuals (obtained from the national COVID-19 registry) by the estimated hospitalization risk among symptomatic cases (derived from international studies). This back-calculation approach allowed us to estimate age-specific infection rates despite limited data. Similar methods have been applied in studies using the US Centers for Disease Control and Prevention COVID-NET data and Health Affairs modeling.^17,18^ We referred to previous studies to estimate the age-specific risks of hospitalization for COVID-19-infected patients during the Omicron era.^19^ Additionally, we estimated the COVID-19 symptomatic infection rates and risk of hospitalization for partially and fully vaccinated individuals by multiplying the vaccine efficacy by the infection rates and risk of hospitalization in non-vaccinated individuals, respectively.

 We obtained data on the probability of ICU admission, mechanical ventilation, death, and length of stay in COVID-19 patients based on their age group and vaccination status. To do so, we analyzed data from 451 262 hospitalized COVID-19 cases, which included 116 001 nonvaccinated cases, 26302 partially vaccinated cases, and 303 959 fully vaccinated cases.^20^ The cases were sourced from the national system of the Medical Care Monitoring Center (MCMC) of the Iranian Ministry of Health and Medical Education (MoHME). We collected this data over 70 days, from January 21 to the end of March 2022, during the country’s sixth hospitalization peak of COVID-19 in the Omicron (B.1.1.529)-dominant era. Patient information, such as name, age, sex, city of residence, clinical signs and symptoms, comorbidities, COVID-19 severity, ICU admission, ventilatory support (noninvasive and invasive), vaccination status, length of stay, and discharge status (death, complete or partial recovery, etc), was recorded in the system. The data were collected from hospitals across the country. We calculated the point estimate and 95% confidence interval for each parameter based on age group and vaccination status (Table S1).

 The MCMC registry operates under standardized national protocols with regular data validation procedures, ensuring completeness and consistency across reporting units. These datasets are routinely used by the Iranian MoHME for surveillance and policy decisions, supporting their credibility.

 Also, the model did not explicitly account for underlying comorbidities such as diabetes or hypertension due to a lack of stratified data on comorbidity-specific outcomes in national registries during the study period. However, the increased severity associated with such conditions was indirectly reflected through higher age-specific hospitalization and mortality rates.

 Although reinfection transitions were not explicitly modeled in the Markov-SIR structure, this uncertainty was addressed by varying the infection rate parameter across a wide, plausible range in the one-way sensitivity analyses. National data of Iran showed very low reinfection rates during the Omicron period, such as 0.03% in ICU patients.^21^ Global reports also indicated reinfection rates mostly under 10%, including 5.18% in Hong Kong,^22^ 9.72% in Mainland China,^23^ and 1.8% in Malaysia,^24^ while a meta-analysis estimated a global prevalence of 4.2%.^25^ Given these low rates and the one-year horizon, excluding reinfection transitions is unlikely to meaningfully affect incremental cost-effectiveness ratio (ICER) estimations. Moreover, sensitivity analyses confirmed that even under the highest infection rate scenarios, vaccination remained highly cost-effective.

####  Vaccine Efficacy and Supply

 We obtained data on vaccination coverage from official websites that provide COVID-19 statistics, as well as from the MoHME in Iran for 2021.^27^ In our model, we assumed that the vaccination coverage rate was the same across all age categories. Vaccine efficacy was defined as the proportional reduction in COVID-19 infections, hospitalizations, and ICU admissions. The Sinopharm vaccine was the primary vaccine used in Iran, accounting for 80% of injections. We relied on the literature to determine its efficacy in preventing COVID-19 cases and hospitalizations during the Omicron (B.1.1.529) wave period.^28-30^ However, we were unable to obtain data on the efficacy of a single dose of the Sinopharm vaccine during our literature review; therefore, we used data on the efficacy of other vaccines belonging to the inactivated vaccine platform for partially vaccinated individuals in our model. According to the literature data on vaccine immunogenicity, we assumed that vaccine efficacy would not decrease over a one-year horizon (Table S2).^13^

####  Costs 

 The cost of treating patients at different stages of infection was calculated by including the costs of outpatient visits and treatment, general hospitalization, ICU admission, ventilator support, and post-discharge costs for patients requiring ICU admission and mechanical ventilation. We derived these costs from health insurance data and hospitalized COVID-19 patient records. We obtained data on the cost of COVID-19 vaccination, including the unit price of COVID-19 vaccine per dose, wastage, and incremental system cost of introduction, from national official websites’ reports regarding the COVID-19 vaccination program in Iran and the literature.^31,32^ Table S3 shows the mean duration of hospitalization only or ICU admission considering the use of a ventilator or not stratified by 3 subgroups of vaccination status (non-vaccinated, partially vaccinated, and fully vaccinated). The length of stay gradually increased every 10 years after 24 years, regardless of the vaccination status.

 The cost of COVID-19 vaccines was treated as a fixed value based on average national procurement reports during the study period. To reflect uncertainty due to potential bulk purchase discounts or future price fluctuations, a plausible cost range was applied in the one-way sensitivity analysis.

 The cost of the Sinopharm vaccine, which accounted for 80% of vaccine injections in Iran’s vaccination program, was used as the index for calculations (Table 1).

**Table 1:** COVID-19 Vaccination Cost, COVID-19 Related Hospitalization Cost, Costs for Hospitalized Patient Requiring ICU + Ventilator

**Variable**		** Mean **	**Lower Limit**	**Upper Limit**
COVID-19 vaccination cost (US$)	Unit price of COVID-19 vaccine (per dose)	9.20	6.00	10.00
	Wastage (US$)	0.46	0.30	0.50
	Incremental system costs of introduction (US$)	1.13	1.02	1.24
	Total cost of vaccination per dose (US$)	10.79	7.32	11.74
Outpatient treatment cost (US$)		19.02	15.21	22.82
COVID-19 related Hospitalization cost (US$)	Hospitalized patient not requiring ICU or ventilator	286.66	267.65	305.68
	Hospitalized patient requiring ICU	650.39	630.98	668.83
	Hospitalized patient requiring ICU + ventilator	797.14	685.55	908.73
After discharged costs for hospitalized patient requiring ICU + ventilator (US$)		94.82	79.61	117.64
Daily wage (US$)		9.51	7.61	13.31
Workforce participation rate (%)	18-24 years	59.10	55.00	65.00
	25-34 years	53.00	50.00	56.00
	≥35 years	46.50	43.00	49.00

Abbreviation: ICU, intensive care unit.

 Productivity losses were estimated by multiplying the average duration of COVID-19 symptoms, including duration for outpatients and length of stay in the hospital, by workforce participation rates and average daily wages. We extracted workforce participation rates for different age groups and average daily wages from the National Statistics Center of Iran websites and entered them into the model (Table 1).^33^ All the costs were converted to US dollars (USD) by using the average exchange rate in 2021 (1 USD = 262 931 IR Rials).

####  Health Utilities

 We obtained utility weights for the normal population in all age categories in Iran by referring to previous national studies on the quality of life in Iran.^34^ We also obtained utility losses for various disease progression states, including symptomatic infection, general hospitalization, and ICU admission with and without mechanical ventilation, from the literature. These utility weights and losses were used in our model to estimate the overall health impact of COVID-19 vaccination (Table 2). Utility weights for different health states were obtained from previously published COVID-19 cost-utility studies and are summarized along with their sources in Table S1.

**Table 2:** Utility Weights for Different Health States

**Variable**		**Mean**	**Lower Limit of ** **95% CI**	**Upper Limit of** **95% CI **
Utility weights for the normal population	18-34 years	0.87	0.83	0.91
	35-44 years	0.83	0.79	0.87
	45-54 years	0.78	0.74	0.82
	55-64 years	0.75	0.71	0.79
	65-75 years	0.74	0.70	0.78
	>75 years	0.67	0.64	0.70
Detected infection symptoms disutility weight		0.19	0.17	0.21
Detected infection hospitalization as the highest setting disutility weight		0.30	0.27	0.33
Detected infection hospitalization with ICU as the highest setting disutility weight		0.50	0.45	0.55
Detected infection hospitalization with ICU + ventilator as the highest setting disutility weight		0.60	0.54	0.66

Abbreviations: ICU, intensive care unit; CI, confidence interval. Note: All utility values are presented on a scale from 0 (equivalent to death) to 1 (full health). Disutility values ranged from 0 (representing no disutility or the state is neutral or desired) and 1, representing maximum disutility.

###  Base Case Analysis

 Using these utility weights and losses, we were able to estimate the overall health impact of COVID-19 vaccination in our model. Specifically, we calculated the number of COVID-19 symptomatic cases, hospitalizations, ICU admissions, and deaths averted over one year, as well as the corresponding direct healthcare and productivity costs saved and QALYs gained. Our analysis was conducted from a societal perspective. Furthermore, we determined the incremental costs and incremental QALYs for the vaccination strategy compared with no vaccination. To assess the cost-effectiveness of the vaccination strategy, we calculated the ICER, which represents the incremental cost per QALY gained. Our willingness-to-pay (WTP) threshold for ICER was set to <US$4091.

 The selection of input parameters, summarized in Table S1, was based on a combination of data sources. Whenever available, we used real-world national data from the MCMC and the MoHME. For parameters lacking local data, values were extracted from published literature, prioritizing studies conducted in Middle Eastern or LMIC settings. In cases where neither local data nor reliable literature was available, parameter estimates were informed by expert consensus from national epidemiologists and health economists. All sources are documented in the supplementary materials.

 Vaccine efficacy was assumed to remain constant throughout the one-year modeling period. While this assumption does not account for potential waning immunity, a wide range of efficacy values was tested in one-way sensitivity analyses to capture this uncertainty.

###  Sensitivity Analysis

 In one-way sensitivity analysis, one input (such as vaccine efficacy or cost) is varied at a time, while all other parameters are held constant. This approach identifies which inputs have the greatest influence on the model outcomes, particularly the ICER. To address uncertainty in the model, one-way sensitivity analyses were performed by varying each parameter individually across a predefined plausible range obtained from published literature or expert input. The 10 most influential parameters were identified based on how variations in their values affected the ICER. These parameters were visually represented in a tornado diagram. Due to the lack of reliable distributional data for several key inputs and the limited one-year time horizon, probabilistic sensitivity analysis (PSA) using Monte Carlo simulation was not conducted. This limitation has been acknowledged, and PSA is recommended for future models with richer datasets and extended projection periods. Given the one-year time horizon of the present study, the impact of discounting on the results was minimal; therefore, assigning a distribution to the discount rate in sensitivity analyses was not deemed necessary.

 Although not performed in this study, future analyses could benefit from incorporating PSA using Monte Carlo simulation to better capture the joint uncertainty across multiple model parameters, particularly in longer-term projections or more complex policy evaluations. Additionally, reinfection rates and variant-specific severity were not modeled explicitly due to data limitations, their potential effects were indirectly addressed by varying key parameters such as infection rate, hospitalization risk, and case fatality in the one-way sensitivity analyses.

## Results

###  Primary Results

 Over a one-year period, 60 655 600 people were at risk of COVID-19. No vaccination resulted in 2 0317 221 new cases, 1 510 803 hospitalizations, and 135 115 deaths from COVID-19. However, the vaccination program prevented 2 078 176 infections, 833 951 hospitalizations, 87 273 ICU admissions, and 43 161 deaths in all age groups (Table 3). The average cost per individual for the vaccination program was $39.68, while the non-vaccination cost was $25.6 per person. Compared to the non-vaccinating scenario, vaccination resulted in an average of $14.08 more cost per person and produced an average of 0.035 additional QALYs per person. Although vaccination was not cost-saving compared to non-vaccinating, it was still considered a cost-effective strategy due to generating an average of 0.035 incremental QALYs per person. Overall, vaccination generated an extra 2 098 495 QALYs at a total extra cost of $853.78 million. The average incremental cost per QALY saved in the vaccination scenario was $406.85 in the adult population. This value corresponds to the vaccinated population, not the entire national population. For better interpretability, this equates to approximately 3500 QALYs per 100 000 vaccinated individuals. Based on one-way sensitivity analysis, these three parameters, vaccine cost per dose, hospitalization risk in unvaccinated individuals, and vaccination coverage among individuals aged 18–64, produced the largest variations in ICER values across all age-specific models. These results were derived from the tornado diagram, which ranked parameters by their influence on the outcome.

**Table 3:** Calculation of Average Cost, Incremental Cost, Average QALY, Incremental QALY, and Incremental Cost-Effectiveness Ratio of COVID-19 Vaccines in Iran in Different Age Groups of Non-vaccinated and Vaccinated COVID-19 Patients

**Vaccination Status**	**Age Group (y)**													
	**Above 64 **		**55-64 **		**45-54**		**35-44**		**25-34 **		**18-24 **		**All Age**	
	**Non-Vaccinated**	**Vaccinated**	**Non-Vaccinated**	**Vaccinated**	**Non-Vaccinated**	**Vaccinated**	**Non-Vaccinated**	**Vaccinated**	**Non-Vaccinated**	**Vaccinated**	**Non-Vaccinated**	**Vaccinated**	**Non-Vaccinated**	**Vaccinated**
Population at risk	5 691 000	5 691 000	6 968 000	6 968 000	10 192 000	10 192 000	15 548 000	15 548 000	14 541 000	14 541 000	7 715 600	7 715 600	60 655 600	60 655 600
COVID-19 infections	1 938 678	1 740 596	3 188 986	2 889 377	3 621 753	3 254 072	4 756 744	4 258 029	4 449 854	3 983 320	2 361 207	2 113 652	20 317 221	18 239 046
Hospitalizations	350 901	157 213	207 284	93 719	235 414	105 548	294 918	131 738	275 891	123 239	146 395	65 394	1 510 803	676 851
ICU admission	67 022	25 970	23 216	8370	17 656	5520	12 976	3904	8829	2584	5417	1495	135 115	47 843
COVID-19 deaths	41 194	14 289	9684	3074	6947	1907	4106	1062	1324	414	845	196	64 101	20 940
Decreased infections		198 082		299 609		367 681		498 715		466 534		247 555		2 078 176
Decreased hospitalizations		193 688		113 565		129 866		163 180		152 652		81 001		833 951
Decreased ICUs		41 052		14 846		12 136		9073		6244		3922		87 273
Decreased deaths		26 905		6610		5041		3045		911		649		43 161
Average cost (US$)	40.41	45.86	33.00	45.64	25.20	39.67	21.11	36.73	22.37	37.90	23.71	39.05	25.60	39.68
Incremental cost (US$)		5.45		12.64		14.47		15.62		15.54		15.34		14.08
Average QALY	4.32	4.37	8.18	8.21	10.99	11.03	12.89	12.92	14.43	14.46	15.43	15.47	11.92	11.95
Incremental QALY		0.047		0.033		0.034		0.034		0.034		0.034		0.035
Incremental cost-effectiveness ratio (US$)		114.92		380.57		429.17		469.51		471.17		461.22		406.85

Abbreviations: ICU, intensive care unit; QALY, quality-adjusted life year.

###  Base Case Results

####  Incremental Benefits of Vaccination in the Age Group of 18-24 Years 

 In the age group of 18-24 years, vaccination prevented a total of 247 555 infections (11.9% of the total adult population prevented), 81 001 hospitalizations, 3922 ICU admissions, and 649 deaths over one year. However, this group had the lowest hospitalizations averted (9.71% of the total prevented hospitalizations), ICU admissions averted (4.49% of the total adult population prevented ICU admissions), and death cases averted (1.5% of the total population’s prevented deaths) compared to other age groups.

 The incremental cost for vaccinating individuals over 64 years was the lowest at $5.45, while those under 64 years had an incremental cost ranging between $12.64 and $15.54. The average incremental cost for this age group was $15.34, with an average gain of 0.033 QALYs per person. The total ICER for this group was $461, which was higher than the average ICER for all adult populations.

 An average $15.34 incremental cost was incurred by vaccinating this age group, and an average of 0.033 QALY was gained per person. The total ICER was $461 for this group, which was higher than the average ICER for the adult population.

####  Incremental Benefits of Vaccination in the Age Group of 25-34 Years 

 Compared to other age groups, the vaccination program yielded the lowest cost-effectiveness in the 25-34 age group, with an ICER of $471 per QALY generated. This age group accounted for 22.44% of the total prevented infections (466 534 cases), 18.30% of prevented hospitalizations (152 652 cases), 7.15% of prevented ICU admissions (6244 cases), and 2.11% of prevented deaths (911 cases) due to vaccination.

####  Incremental Benefits of Vaccination in the Age Group of 35-64 Years 

 The age group with the most infections averted was 35-44 years old, with 498 715 cases averted. In contrast, the age group over 64 years only accounted for 9.53% of the total averted new infections in the adult population. Table 3 provides detailed information on the incremental benefits of vaccination for people aged 45 to 64 years old.

####  Incremental Benefits of Vaccination in the Age Group of Over 64 Years 

 Vaccination prevented a total of 198 082 new infections, 193 688 hospitalizations, 41 052 ICU admissions, and 26 905 deaths in individuals over the age of 64 years. The ICER was lowest for this age group and increased with each decade below 64 years until reaching a peak at 25-34 years old. Among all age groups, vaccination was most cost-effective and effective in individuals over 64 years old. This age group gained the most incremental QALYs and accounted for the highest number of prevented hospitalizations (193 688) with the least incremental cost ($5.45). However, vaccination had the least impact on preventing infections in individuals over 64 years old, with only 198 082 cases averted. This age group also had the most prevented ICU admissions (41 052) and deaths due to vaccination. Overall, vaccination was more cost-effective in individuals over 64 years old compared to other age groups.

**Figure 2:**
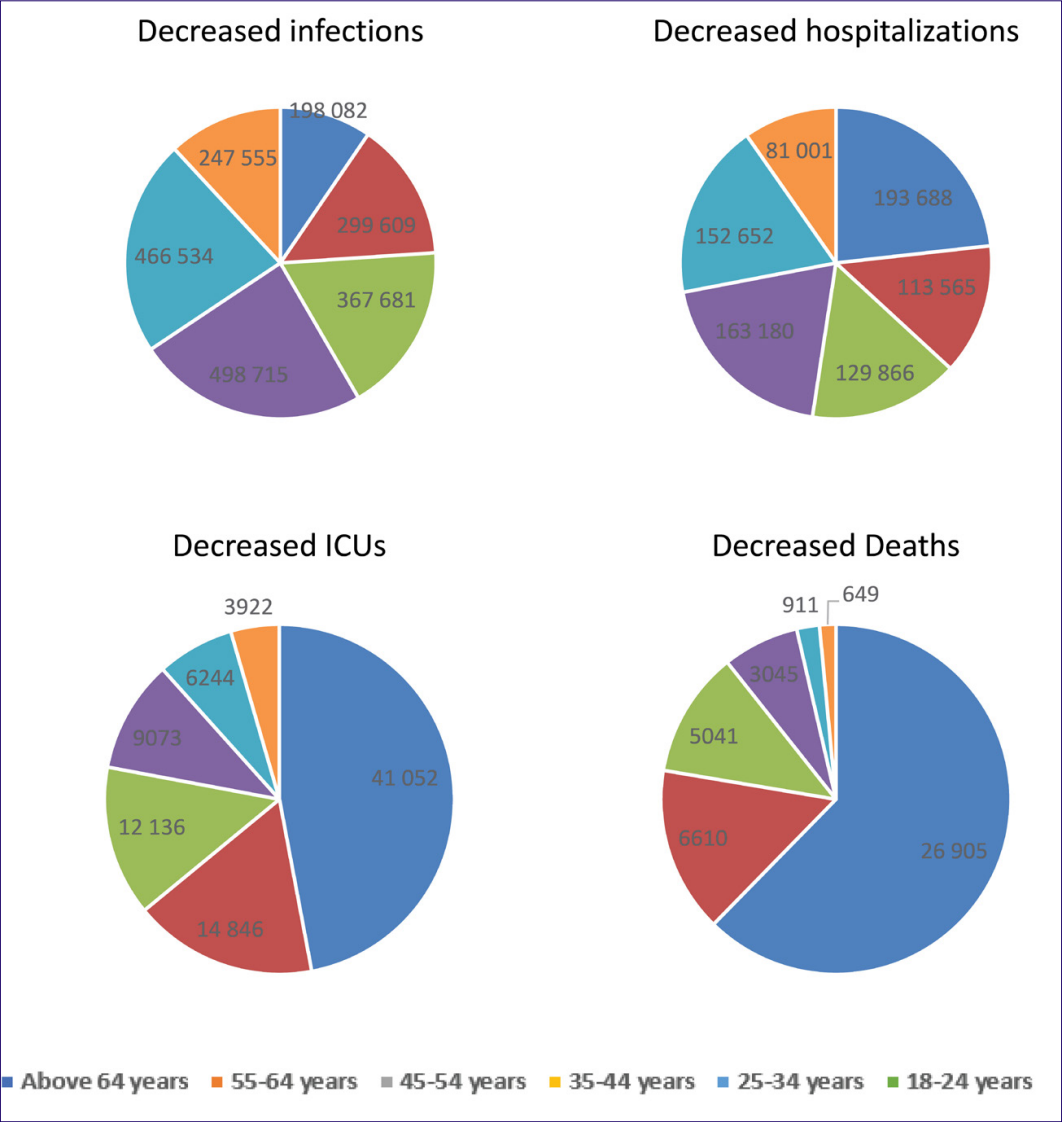
The Incremental Benefits of Decreased COVID-19 Infections Cases and Severe Clinical Outcomes of the COVID-19 Vaccination Strategy in Varied Age Groups in Iran. The figure shows a comprehensive overview of the incremental benefits of vaccination across age groups, including reductions in infection, hospitalization, intensive care unit admissions, and deaths. The aim was to provide a how outcomes vary by age. Abbreviation: ICU, intensive care unit.

Figure 2 demonstrates the incremental benefits of decreased COVID-19 infection cases and severe clinical outcomes of the COVID-19 vaccination strategy in various age groups.

###  Sensitivity Analyses

 The cost of the COVID-19 vaccine per dose, risk of hospitalization in non-vaccinated patients, and coverage of vaccination in individuals aged 18-64 years were the most sensitive parameters in all age-based sensitivity analyses. For those over 64 years old, the most sensitive parameters were the cost of COVID-19 vaccine per dose, COVID-19 symptomatic infection rate, and risk of hospitalization in non-vaccinated COVID-19 patients.

 The cost of vaccine per dose was the most influential parameter affecting ICER, with an increase in cost leading to an increase in ICER. The ICER varied widely when vaccine cost changed within a range of $7.32 to $11.74 per injection. Vaccination was even cost-saving for those over 64 years old when the vaccine price was at its lowest value. The highest ICER value was observed in those aged 25 to 35 years, with a vaccination cost of $525.

**Figure 3:**
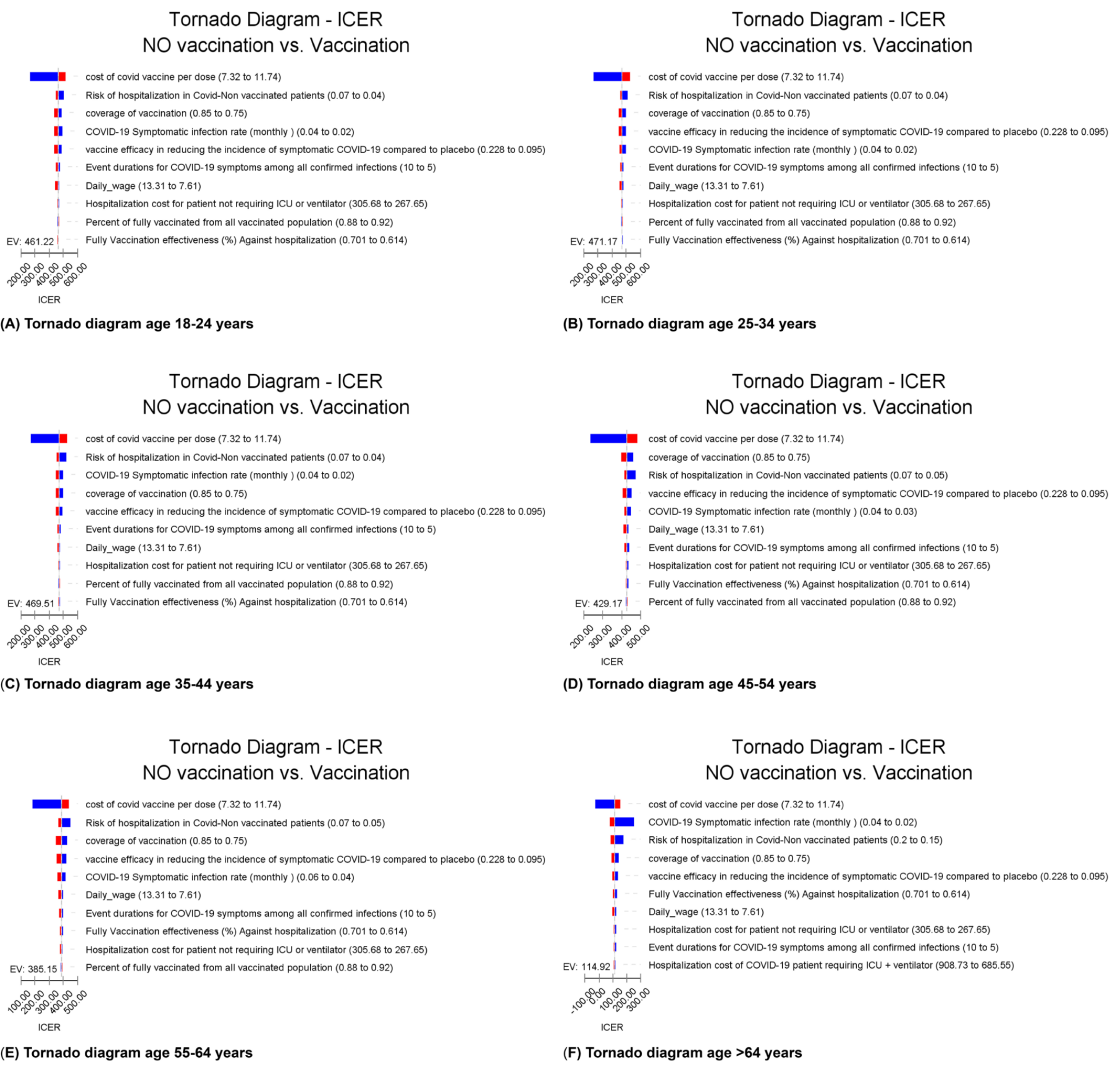
Sensitivity Analysis: Tornado Diagram A to F According to Different Age Groups. Tornado diagram showing the impact of the sensitivity analyses on the incremental cost per quality-adjusted life-year gained of vaccination compared to no vaccination. A horizontal bar was generated for each parameter analysis. The width of the bar indicates the potential effect of the associated parameter on the ICER when the parameter is changed within its range. The red part of each bar indicates high values of input parameter ranges, while the blue part indicates low values. Abbreviations: ICER, incremental cost-effectiveness ratio; ICU, intensive care unit.

 A higher risk of hospitalization in non-vaccinated COVID-19 patients and increased vaccination coverage decreased the ICER. For those over 64 years old, higher symptomatic COVID-19 infection rates decreased the ICER. All results were robust in sensitivity analysis, with vaccination remaining cost-effective even after changing parameter ranges. The Tornado diagrams are available in Figures 3A to 3F.

## Discussion

 In this study, we assessed the cost-effectiveness of the COVID-19 mass vaccination program during the Omicron era in Iran. Our results indicated that the vaccination strategy significantly reduced the incidence of infections, hospitalizations, ICU admissions, and deaths among adults in all age groups when compared to no vaccination. The vaccination program averted a total of 2 078 176 infections, 833 951 hospitalizations, 87 273 ICU admissions, and 43 161 deaths across all age groups. The number of deaths prevented ranged from 649 deaths in the 18-24 age group to 26 905 deaths in those over 64 years.

 The cost-effectiveness threshold is the maximum amount that decision-makers are willing to pay for a unit of health outcome based on the country’s gross domestic product (GDP) per capita.^35^ The 25 ICER threshold for cost-effectiveness was 4091 (1 time GDP per capita) per QALY. The average incremental cost per QALY saved in the vaccination scenario was 406.85 per QALY in adult people regardless of their age group, which is about 0.16 times the cost-effectiveness threshold in Iran.

 The vaccination strategy resulted in a gain of 2 098 495 QALYs during a one-year period. The ICER was lowest for those over 64 years old and increased by every 10 years below 64 years old until 25 years old. Vaccination was found to be a highly cost-effective intervention in all population groups. Additionally, our results were robust in an extensive sensitivity analysis. These findings are consistent with evidence from other countries that found vaccination to decrease COVID-19 infection and death compared to a no-vaccination strategy and increase clinical benefits.^10,36-39^ However, an important difference between our study and others is that our cost-effectiveness analyses were based on real data after a mass vaccination program rather than prospective simulations before vaccination.

 Vaccination in patients over 64 years brought the most cost-effectiveness among all age groups of the population, accounting for the higher proportion of decreased infections, hospitalizations, ICU admissions, and deaths, and accordingly, a lower value of ICER, as we found the ICER was the lowest for the elderly age group. Therefore, vaccinating this population is likely to be considered a great priority.^13^ Several studies have agreed that prioritizing vaccination for the elderly population brings more effectiveness and could be even cost-saving compared to the all-age vaccination strategy. A study conducted by Orangi et al in Kenya showed because of the low proportion of the elderly population in Kenya (11% of the total population is aged 50 years and above), prioritizing them in the vaccination strategy was highly cost-effective, even with low vaccine coverage (only 30% coverage), while the expansion of vaccination to whole population needed great vaccine coverage to achieve cost-effectiveness.^40^ Elderly people are at higher risk of contracting severe forms of infection and have a higher chance of increasing comorbidities leading to severe infection complications, hospitalization, ICU admissions, and deaths.^13^ Moreover, the highest values of direct health costs belonged to the patients who were admitted to the ICU and needed mechanical ventilation. Thus, preventing infection and severe disease in the elderly would bring a high cost-effectiveness as they account for the highest proportion of hospitalizations and deaths among all age groups of the population. A study conducted by Li et al extensively evaluated the cost-effectiveness of a BNT162b2 booster strategy among older adults aged ≥65 years in the United States and demonstrated that with every dollar of investment in the booster vaccination, the US government might save nearly $2 because of fewer COVID-19 hospitalizations. Notably, the cost-effectiveness of the booster strategy is highly sensitive to the incidence of COVID-19, consistent with our results, which showed that the more the risk of death and hospitalization increased, the more cost-effective the vaccination program.^14^

 Studies conducted in the UK, the US, and Madagascar have accordingly shown that prioritizing older ages and taking the proportion of the older population into account based on population demographics could bring better cost-effectiveness in different regions.^36,38-40^ In contrast to previous studies highlighting the cost-effectiveness of prioritizing vaccination for the elderly population, Pearson et al conducted a study in Pakistan that found unprioritized vaccination would bring greater benefits. This is attributed to the demographic context of LMICs, which have a younger population and higher previous infection rates compared to high-income countries.^10,41^ Additionally, vaccines that only protect against the disease rather than infection transmission have even lower benefits in these settings. Similar results were observed in a study in South Africa, indicating that achieving cost-effectiveness when targeting the entire population will require high coverage of vaccination in settings with a lower proportion of elderly individuals and a higher probability of previous exposure to COVID-19.^10,37,40^ The young population is actively working and has high-transmission tendencies; therefore, vaccinating them with even less effective vaccines but a high coverage would decrease the transmission of infection in the whole population and decrease the probability of infection in older ages, indirectly protecting this vulnerable group.^10,42^ A better cost-effectiveness profile would have been achieved when taking productivity losses into account from a societal perspective rather than a health system’s perspective, which considers only direct health system costs; As observed in the studies of Turkey and Pakistan, taking into account productivity losses even made the vaccination program cost-saving rather than just cost-effective, while the productivity losses were not considered.^10,40,43^ These findings mirror the results of our study. The young population has accounted for a high proportion of the population in Iran. At the same time, a greater proportion of infections averted with vaccination belongs to the population aged 18-45 years, accounting for 73% of total infections averted in the adult population through vaccination. Although we found the incremental cost per QALY generated was highest in ages 18-45 years, and they developed a lower proportion of averted hospitalizations, ICU admissions, and deaths after vaccination compared to the elderly population, vaccinating this population with a high coverage still brought a high beneficial value as the ICER was still far from reaching the cost-effectiveness threshold. The reason is that the young population has a potentially lower overall risk of developing severe infection leading to hospitalization and death and is more likely to get mild and or asymptomatic COVID-19 infection. Moreover, they have a higher chance of being previously exposed to the infection and developing natural immunity. Nevertheless, as they are active working groups with higher value of productivity, preventing them from being infected not only decreases the costs indirectly incurred on the economy but also indirectly protects the vulnerable population by averting severe infections leading to hospitalization and death.

 Based on our findings, vaccination was not cost-saving compared to the non-vaccination scenario in any age group. It could have several reasons. In many studies, the effectiveness of the vaccines was considered in the period of early strains of the virus, but in this study, the effectiveness of the vaccine was based on the extracted real data in the Omicron period with lower effectiveness because of decreased pathogenicity, rate of hospitalization, and death. Furthermore, we did not estimate the direct influence of infection transmission reductions by vaccination among the population individuals, and the real beneficial effect could be greater than what we roughly calculated.^13^ Besides, in Iran, the costs of hospitalization and treatment are much lower than in Western countries due to lower wage rates and the existence of hidden subsidies in the economy, while the price of vaccines is similar to other countries. Thus, the vaccination program and reducing the cost of treatment in other countries lead to much more profit compared to Iran. While comparable cost-effectiveness studies in other LMICs often support prioritization of elderly populations due to higher mortality risk, Iran’s relatively younger demographic structure poses different policy considerations. Our model demonstrated that vaccination among adults aged 18–64 years was highly cost-effective, largely due to their higher representation in the workforce and their role in transmission. Therefore, age-based prioritization strategies should be context-specific, taking into account both clinical risk and demographic realities.^44^ This study relied on a combination of real-world data for the vaccination scenario and modeled assumptions for the counterfactual non-vaccination scenario. While this approach allows for practical relevance, it introduces inherent uncertainty in comparative outcomes. Moreover, unlike some LMIC-based studies that have reported COVID-19 vaccination to be cost-saving, our findings indicated cost-effectiveness but not universal cost-saving. This discrepancy may be attributed to Iran’s lower healthcare costs, younger population structure, and limited treatment expenditures relative to other countries. These differences highlight the importance of contextualizing economic evaluations within national health and cost frameworks.

 In this study, we only calculated the benefits of preventing productivity loss without considering suppression policies. These policies enforced by the governments had substantial net benefits in terms of preventing losses to economic output; on the other hand, suppression policies resulted in substantial losses to the GDP, too. In total, the net benefits of suppression policies on total economic production were positive and likely substantial. In contrast, Jiang et al analyzed the cost-effectiveness of vaccinating 50% of the population in Hong Kong special administrative regions, Indonesia, mainland China, Philippines, Singapore, and Thailand, suggesting that the vaccination strategy was not only cost-effective but also created sizeable net monetary benefit. Whereas the deterministic results were robust to parameter variations, the magnitude of net monetary benefit increased with the incidence of COVID-19 and the population coverage of vaccines.^45^

 According to the WHO, interventions that cost less than 3 times GDP per capita are cost-effective, while those that cost less than 1 times GDP per capita are highly cost-effective.^41^ Based on this, vaccination was highly cost-effective in all age groups of the adult population in this study. None of the imputed parameters could change the conclusion that vaccination was a cost-effective intervention in the adult population at risk of COVID-19. The highest value of ICER extracted from the different ranges of imputed parameters in the sensitivity analysis was far below the WTP threshold, which was equal to 1 GDP per capita in our study. Changing the model parameters’ values in their range did not alter the conclusion on the cost-effectiveness of vaccination strategies, and consistent results with the base analyses were obtained from sensitivity analyses. The robust result of the sensitivity analyses brings certainty about model inputs.^41^ Results of the sensitivity analyses showed that the price of the vaccine per dose was the most influential parameter to determine the value of the incremental cost-effectiveness of vaccination. The ICER value varied widely when the price of the vaccine changed in its range, making this parameter the most sensitive factor to determine the cost-effectiveness of the vaccination program. Vaccination with low-priced vaccines was even cost-saving in over 64 years, according to our analysis. Consistent with our result, in a systematic review of studies on the cost-effectiveness of COVID-19 vaccines in multiple countries, one of the most important parameters in determining the cost-effectiveness of the vaccination program was vaccine cost.^46^ In the sensitivity analyses of 12 large LMICs, the vaccination program cost was the most influential factor in determining cost-effectiveness.^47^ Although vaccination has been a cost-effective or cost-saving strategy in both high and LMICs, due to the limited resources, the affordability of vaccines is an important aspect of the mass vaccination program, and the same vaccines and doses cannot be supplied in all countries.^10,41,46^ For low per-dose vaccine prices, either prioritized or non-prioritized strategies would be cost-effective, while high-price vaccines may not be cost-effective depending on their characteristics, vaccination strategy, and pandemic trends.^10^ Other influential parameters were the risk of hospitalization in non-vaccinated COVID-19 patients, vaccination coverage, and vaccine efficacy in reducing symptomatic infection. We concluded that vaccine prices, hospitalization costs, and vaccine efficacy were the important parameters evaluated in sensitivity analyses of different studies.

 This study has several strengths. First, it was a national study that utilized a combination of real data and modeling rather than relying solely on assumptions. The study included all adult age groups and assessed the effects of different vaccine platforms, including fully and partially vaccinated individuals, as well as those with no vaccine status. The study also evaluated several outcomes, such as the length of hospital or ICU stay and mortality.

 However, it is important to interpret these findings within the context of certain limitations. The SIR-Markov model used in this study cannot predict future epidemics or new variants of COVID-19. Therefore, it is important to consider all available resources when interpreting these findings for public health initiatives related to vaccination and epidemic management. The study did not take into account important underlying diseases, such as diabetes and hypertension, nor did it examine different vaccine platforms. Additionally, we assumed that no reinfection occurs within the one year. We acknowledge that this assumption may oversimplify COVID-19 dynamics, particularly with emerging variants that could influence reinfection rates. Reinfection was considered a secondary factor, as initial vaccine effectiveness is generally high and immunity decay in the first year is gradual. Incorporating reinfection would add complexity, but its impact on the model’s conclusions would be limited within the one-year timeframe. Notably, national studies’ evidence has indicated a low recurrent infection rate in Iran during one year.^20^ We assumed that vaccine efficacy would not decrease over a one-year horizon. While this assumption may slightly affect the ICER, the impact is expected to be unremarkable, as our primary focus was on short-term health outcomes and economic benefits. Extending the model timeframe or projecting further could require incorporating waning immunity more comprehensively, particularly in light of booster requirements and emerging variants. The study did not assess the long-term complications of COVID-19 and social outcomes of the COVID-19 pandemic, which could further increase the economic burden of the disease when not vaccinating, as well as the limitation of critical care capacity during the peak of the Omicron COVID-19 outbreak. On the other hand, COVID-19 vaccine complications could be simultaneously considered. Finally, the study did not consider patient overcrowding or critical care capacity during the peak of the Omicron COVID-19 outbreak, which may underscore the importance of mass vaccination, especially in LMICs with large populations. Given the one-year time horizon and national surveillance data indicating low reinfection rates during the Omicron wave in Iran, transitions from recovered to susceptible were excluded. While this may underestimate the true transmission dynamics, one-way sensitivity analyses incorporating a wide range of infection rates were conducted to account for the potential effects of reinfection. Therefore, this limitation is unlikely to significantly affect the overall conclusions of the model.

 Another methodological limitation is the application of a 3% discount rate, which, although consistent with international guidelines, may not reflect Iran’s economic environment. Given the one-year model horizon, this had a limited impact, but future evaluations with longer timeframes should consider locally appropriate rates. The model did not incorporate waning vaccine immunity over time, which may overestimate long-term protection. However, sensitivity analyses involving a range of efficacy values partially addressed this limitation. More dynamic modeling approaches are recommended for studies with longer time horizons or booster dose evaluations. Another limitation is that vaccine effectiveness was modeled as a single averaged value without differentiating between vaccine types. Although Sinopharm accounted for the majority of doses administered, this approach may not capture potential variability in protection across different vaccine brands. The findings of this study are based on epidemiological and healthcare data from Iran during the Omicron wave and may not be generalizable to other variants with different transmission dynamics or to countries with different healthcare infrastructures and vaccine coverage. Caution should be taken when extrapolating these results to other settings without appropriate model recalibration. Also, the model did not incorporate critical care capacity constraints during COVID-19 surges, such as ICU bed shortages. This simplification may underestimate the full benefits of vaccination in preventing hospital overload, treatment delays, and excess mortality caused by strained healthcare resources.

 While the analysis incorporated key healthcare and individual-level societal costs, it did not extend to capturing broader macroeconomic consequences of the pandemic, such as national GDP fluctuations, lockdown-induced business closures, or large-scale economic slowdowns. As a result, the estimated economic benefit of vaccination may represent a conservative approximation of its total value to society. Although this analysis focused on economic outcomes, equity and feasibility issues remain important. The model did not assess disparities in vaccine access or challenges in achieving high coverage in resource-limited settings. Real-world constraints such as logistical barriers and vaccine hesitancy may limit the impact of vaccination programs and should be considered in policy planning. The exclusion of reinfection may lead to a slight underestimation of the total disease burden; however, given the low observed reinfection rates during the study period, its impact on the economic outcomes and overall cost-effectiveness conclusions is likely negligible.

## Conclusion

 In summary, cost-effectiveness analysis plays a fundamental role in decision-making and the implementation, evaluation, and monitoring of public policies. Our epidemiological approach for assessing the impact of vaccination strategies against COVID-19 constitutes a tool that articulates scientific knowledge, empirical evidence, and public policies in a framework for interaction with users, analysts, and decision-makers. Expanding the vaccination strategy to eligible individuals would be cost-effective, as it would result in a significant gain of QALYs by increasing some extra costs. Therefore, the COVID-19 vaccination program was health-beneficial and highly cost-effective in Iran.

## Acknowledgments

 The authors would like to express their gratefulness to the staff and researchers of the Iran MoHME.

## Ethical issues

 National Committee for Ethics in Biomedical Research of Iran had declared a prior mandate that all COVID-19 patients admitted to any medical facility must be made fully aware of their potential participation to research projects. All patients were requested to sign an informed consent form which contained the permission to transfer their electronic data, confidentially, to the hospital information system which are connected to the national registry of the MCMC of the Iranian Ministry of Health for data collection and publication, future investigations, and research.

 This study was approved by the Iran National Committee for Ethics in Biomedical Research (Ethic code: IR.SBMU.NRITLD.REC.1402.088) and followed the ethical guidelines of the Declaration of Helsinki.

## Conflicts of interest

 Authors declare that they have no conflicts of interest.

## Data availability statement

 The data that support the findings of this study are available from the corresponding author upon request.

## Supplementary files


Supplementary file 1 contains Tables S1-S3.
