# Supplementary material for: Assessing the Economic Benefit of Mass COVID-19 Vaccination Program in Iran: A Real-World Modeling Study
Source: Int J Health Policy Manag. 2025 Oct 11;14:8852. doi: 10.34172/ijhpm.8852 (PMC12958171; doi:10.34172/ijhpm.8852)
Supplement: Supplementary file 1 — contains Tables S1-S3. [file ijhpm-14-8852-s001.pdf]

Article title: Assessing the Economic Benefit of Mass COVID-19 Vaccination Program in Iran: A Real-World Modeling Study

Journal name: International Journal of Health Policy and Management (IJHPM)

Authors' information: Hamidreza Jamaati[1], Saeed Karimi[2], Yunes Panahi[3], Shahnam Arshi[4], Maryam Hajimoradi[5], Fatemeh Sadat Hosseini-Baharanchi[6], Fariba Ghorbani[7], Seyed Mohsen Zahraei[4], Fatemeh Nouri[5], Ali Akbari Sari[8], Mahshad Goharimehr[8], Abdolreza Mohamadnia[1], Payam Tabarsi[9], Farzaneh Dastan[1], Babak Sharif-Kashani[10], Majid Marjani[9], Farin Rashid Farokhi[11], Seyed Mohammad Reza Hashemian[1], Mostafa Noorizadeh[12,13], Mojtaba Nouhi[14], Katayoun Tayeri[4], Sima Noorali[5], Farnaz Ahmadi[5], Makan Sadr[15], Azadeh Moradkhani[1], Mahdi Ahmadinia[5], Bahamin Astani[5], Rajabali Daroudi[8]*, Shadi Shafaghi[5]*

[1]Chronic Respiratory Disease Research Center, National Research Institute of Tuberculosis and Lung Disease, Shahid Beheshti University of Medical Science, Tehran, Iran.

[2]Department of Ophthalmology, Torfeh Medical Center, Shahid Beheshti University of Medical Sciences, Tehran, Iran.

[3]Chemical Injuries Research Center, Baqiyatallah University of Medical Sciences, Tehran, Iran.

[4]Center for Communicable Disease Control, Ministry of Health and Medical Education, Tehran, Iran.

[5]Lung Transplantation Research Center, National Research Institute of Tuberculosis and Lung Diseases (NRITLD), Shahid Beheshti University of Medical Sciences, Tehran, Iran.

[6]Department of Biostatistics, School of Public Health, Iran University of Medical Sciences, Tehran, Iran.

[7]Tracheal Diseases Research Center, National Research Institute of Tuberculosis and Lung Diseases (NRITLD), Shahid Beheshti University of Medical Sciences, Tehran, Iran.

[8]Department of Health Management, Policy and Economics, School of Public Health, Tehran University of Medical Sciences, Tehran, Iran.

[9]Clinical Tuberculosis and Epidemiology Research Center, National Research Institute for Tuberculosis and Lung Disease (NRITLD), Shahid Beheshti University of Medical Sciences, Tehran, Iran.

[10]Department of Cardiology, Lung Transplantation Research Center, National Research Institute of Tuberculosis and Lung Diseases (NRITLD), Shahid Beheshti University of Medical Sciences, Tehran, Iran.

[11]Chronic Kidney Disease Research Center, Shahid Beheshti University of Medical Sciences, Tehran, Iran.

[12]Department of Pharmaceutical Biotechnology, Faculty of Pharmacy, Bulent Ecevit University, Zonguldak, Turkey.

[13]Department of Biotechnology, Islamic Azad University of Medical Science, Tehran, Iran.

[14]Health Economic Department, School of Medicine, Shahed University, Tehran, Iran.

[15]Virology Research Center, National Research Institute of Tuberculosis and Lung Diseases (NRITLD), Shahid Beheshti University of Medical Sciences, Tehran, Iran.

***Correspondence to:** Rajabali Daroudi; Email: rdraoudi@yahoo.com & Shadi Shafaghi; Email: shafaghishadi@yahoo.com

Citation: Jamaati H, Karimi S, Panahi Y, et al. Assessing the economic benefit of mass COVID-19 vaccination program in Iran: a real-world modeling study. Int J Health Policy Manag. 2025;14:8852. doi:10.34172/ijhpm.8852

Supplementary file 1

Table 1S: Probability of hospitalization, ICU admission, ventilator support, and mortality in hospitalized, hospitalized patient requiring ICU or hospitalized patient requiring ICU + ventilator in non-vaccinated, partially vaccinated, and fully vaccinated COVID-19 patients

Variable	Vaccination status	Age group	Mean	Lower limit of 95% CI	Upper limit of 95% CI
COVID-19 Symptomatic infection rate (Monthly)	Non vaccinated	18-24	0.030		
		25-34	0.030	0.020	0.040
		35-44	0.030	0.020	0.040
		45-54	0.036	0.030	0.040
		55-64	0.050	0.040	0.060
		>=65	0.035	0.020	0.040
Probability of hospitalization among symptomatic patients [17]	Non vaccinated	18-24	0.062	0.040	0.070
		25-34	0.062	0.040	0.070
		35-44	0.062	0.040	0.070
		45-54	0.065	0.050	0.070
		55-64	0.065	0.050	0.070
		>=65	0.181	0.150	0.200
Probability of ICU admission in hospitalized patients	Non vaccinated	18-24	0.027	0.024	0.030
		25-34	0.024	0.022	0.026
		35-44	0.032	0.030	0.035
		45-54	0.052	0.048	0.056
		55-64	0.077	0.073	0.082
		>=65	0.128	0.125	0.132
	Partially vaccinated	18-24	0.013	0.010	0.017
		25-34	0.012	0.010	0.015
		35-44	0.017	0.013	0.020
		45-54	0.040	0.033	0.047
		55-64	0.069	0.059	0.079
		>=65	0.116	0.106	0.125

	Fully vaccinated	18-24	0.008	0.007	0.009
		25-34	0.009	0.009	0.010
		35-44	0.014	0.013	0.015
		45-54	0.025	0.024	0.027
		55-64	0.054	0.052	0.056
		>=65	0.107	0.105	0.109
Probability of ICU admission + ventilator in hospitalized patients	Non vaccinated	18-24	0.010	0.008	0.012
		25-34	0.008	0.007	0.009
		35-44	0.012	0.010	0.013
		45-54	0.023	0.021	0.026
		55-64	0.035	0.032	0.038
		>=65	0.063	0.060	0.065
	Partially vaccinated	18-24	0.004	0.002	0.006
		25-34	0.004	0.002	0.005
		35-44	0.004	0.002	0.006
		45-54	0.013	0.009	0.017
		55-64	0.015	0.010	0.020
		>=65	0.049	0.043	0.056
	Fully vaccinated	18-24	0.002	0.001	0.002
		25-34	0.002	0.002	0.003
		35-44	0.003	0.002	0.003
		45-54	0.005	0.005	0.006
		55-64	0.014	0.013	0.015
		>=65	0.033	0.032	0.035
Probability of mortality in hospitalized patient not requiring ICU or ventilator	Non vaccinated	18-24	0.001	0.001	0.002
		25-34	0.001	0.001	0.002
		35-44	0.004	0.003	0.005
		45-54	0.008	0.007	0.010
		55-64	0.014	0.012	0.015
		>=65	0.045	0.043	0.047

	Partially vaccinated	18-24	0.000	0.000	0.000
		25-34	0.001	0.000	0.001
		35-44	0.001	0.000	0.002
		45-54	0.006	0.003	0.009
		55-64	0.010	0.006	0.014
		>=65	0.028	0.023	0.034
	Fully vaccinated	18-24	0.000	0.000	0.001
		25-34	0.001	0.000	0.001
		35-44	0.001	0.001	0.001
		45-54	0.002	0.002	0.003
		55-64	0.006	0.005	0.007
		>=65	0.021	0.020	0.023
Probability of mortality in hospitalized patients requiring ICU	Non vaccinated	18-24	0.056	0.028	0.085
		25-34	0.027	0.012	0.041
		35-44	0.097	0.074	0.120
		45-54	0.147	0.122	0.173
		55-64	0.158	0.137	0.179
		>=65	0.269	0.257	0.282
	Partially vaccinated	18-24	0.021	0.001	0.061
		25-34	0.012	0.001	0.036
		35-44	0.031	0.001	0.065
		45-54	0.098	0.048	0.149
		55-64	0.088	0.046	0.131
		>=65	0.230	0.194	0.267
	Fully vaccinated	18-24	0.020	0.001	0.039
		25-34	0.032	0.017	0.047
		35-44	0.059	0.042	0.075
		45-54	0.078	0.063	0.093
		55-64	0.113	0.100	0.126
		>=65	0.200	0.191	0.208

Probability of mortality in hospitalized patients requiring ICU + ventilator	Non vaccinated	18-24	0.330	0.231	0.428
		25-34	0.398	0.323	0.472
		35-44	0.583	0.519	0.647
		45-54	0.629	0.578	0.681
		55-64	0.632	0.591	0.672
		>=65	0.739	0.722	0.757
	Partially vaccinated	18-24	0.214	0.000	0.429
		25-34	0.458	0.259	0.658
		35-44	0.522	0.318	0.726
		45-54	0.705	0.570	0.839
		55-64	0.649	0.495	0.802
		>=65	0.843	0.794	0.891
	Fully vaccinated	18-24	0.208	0.093	0.323
		25-34	0.366	0.284	0.449
		35-44	0.416	0.343	0.490
		45-54	0.575	0.514	0.636
		55-64	0.627	0.588	0.665
		>=65	0.771	0.756	0.787
Probability of mortality from other causes		Age specific	life table (44)		

Table 2S: Vaccine efficacy in reducing the incidence of symptomatic COVID-19, reducing hospitalization of symptomatic COVID-19 patients in fully and partially vaccination

Variable		Mean	Lower	Upper
Coverage of vaccination (%)	All age	80.00	75.00	85.00
Fully vaccinated from all vaccinated population (%)	All age	90.00	88.00	92.00
Vaccine efficacy in reducing the incidence of symptomatic COVID-19 (%)	Fully Vaccination	16.40	9.50	22.80
	Partially Vaccination	5.00	2.00	7.00
Vaccine efficacy in reducing Hospitalization of symptomatic COVID-19 patients (%)	Fully Vaccination	66.00	61.40	70.10

Table 3S: Length of stay in hospitalized patients not requiring ICU or ventilator, hospitalized patient requiring ICU, and hospitalized patient requiring ventilator in ICU in different age groups of non-vaccinated and vaccinated COVID-19 patients

Variable	Vaccination status	Age group	Mean	Lower	Upper
Length of stay in hospitalized patient not requiring ICU or ventilator	Non vaccinated	18-24	4.160	4.115	4.205
		25-34	4.150	4.116	4.184
		35-44	4.310	4.270	4.350
		45-54	4.420	4.371	4.469
		55-64	4.560	4.503	4.617
		>=65	4.750	4.736	4.764
	Partially vaccinated	18-24	4.230	4.176	4.284
		25-34	4.190	4.147	4.233
		35-44	4.270	4.222	4.318
		45-54	4.350	4.262	4.438
		55-64	4.440	4.328	4.552
		>=65	4.600	4.487	4.713
	Fully vaccinated	18-24	4.230	4.216	4.244
		25-34	4.200	4.189	4.211
		35-44	4.260	4.248	4.272
		45-54	4.290	4.274	4.306
		55-64	4.330	4.307	4.353
		>=65	4.470	4.447	4.493
Length of stay in hospitalized patient requiring ICU	Non vaccinated	18-24	5.100	4.311	5.889
		25-34	4.660	4.161	5.159
		35-44	4.760	4.354	5.166
		45-54	5.680	5.280	6.080
		55-64	5.990	5.637	6.343
		>=65	6.430	6.259	6.601
	Partially vaccinated	18-24	5.030	3.712	6.348

		25-34	4.240	3.400	5.080
		35-44	6.240	4.741	7.739
		45-54	5.420	4.384	6.456
		55-64	5.510	4.638	6.382
		>=65	5.930	5.486	6.374
	Fully vaccinated	18-24	4.720	3.794	5.646
		25-34	3.920	3.583	4.257
		35-44	4.510	4.170	4.850
		45-54	4.960	4.678	5.242
		55-64	5.290	5.084	5.496
		>=65	6.080	5.962	6.198
Length of stay in hospitalized patient requiring ICU + ventilator	Non vaccinated	18-24	6.700	5.260	8.140
		25-34	6.530	5.392	7.668
		35-44	7.540	6.408	8.672
		45-54	7.800	6.833	8.767
		55-64	8.270	7.478	9.062
		>=65	8.220	7.898	8.542
	Partially vaccinated	18-24	10.280	3.559	17.001
		25-34	5.750	3.826	7.674
		35-44	6.180	4.308	8.052
		45-54	7.260	5.567	8.953
		55-64	8.370	5.918	10.822
		>=65	8.730	7.590	9.870
	Fully vaccinated	18-24	7.150	5.161	9.139
		25-34	7.910	6.516	9.304
		35-44	6.660	5.542	7.778
		45-54	7.830	6.839	8.821
		55-64	8.350	7.587	9.113
		>=65	8.330	8.028	8.632
Duration of COVID-19 symptoms for outpatients(day)			7.000	5.000	10.000
